# Multiorgan recovery in a cadaver body using mild hypothermic ECMO treatment in a murine model

**DOI:** 10.1186/s40635-023-00534-2

**Published:** 2023-08-04

**Authors:** Nodir Madrahimov, Vitalii Mutsenko, Ruslan Natanov, Dejan Radaković, André Klapproth, Mohamed Hassan, Mathias Rosenfeldt, Florian Kleefeldt, Ivan Aleksic, Süleyman Ergün, Christoph Otto, Rainer G. Leyh, Constanze Bening

**Affiliations:** 1 Department of Thoracic and Cardiovascular Surgery, University Hospital Würzburg, Würzburg, Germany; 2 Department of Cardiothoracic, Transplantation and Vascular Surgery, Hannover Medical School, Hannover, Germany; 3 Institute for Pathology, Julius-Maximilians-Universität Würzburg, Würzburg, Germany; 4 Institute of Anatomy and Cell Biology, Julius-Maximilians-Universität Würzburg, Würzburg, Germany; 5 Department of General, Visceral, Vascular and Pediatric Surgery, University Hospital Würzburg, Würzburg, Germany

**Keywords:** Extracorporeal membrane oxygenation, Cadaver multiorgan preservation, Mild hypothermia, Post-mortem heart recovery

## Abstract

**Background:**

Transplant candidates on the waiting list are increasingly challenged by the lack of organs. Most of the organs can only be kept viable within very limited timeframes (e.g., mere 4–6 h for heart and lungs exposed to refrigeration temperatures ex vivo). Donation after circulatory death (DCD) using extracorporeal membrane oxygenation (ECMO) can significantly enlarge the donor pool, organ yield per donor, and shelf life. Nevertheless, clinical attempts to recover organs for transplantation after uncontrolled DCD are extremely complex and hardly reproducible. Therefore, as a preliminary strategy to fulfill this task, experimental protocols using feasible animal models are highly warranted. The primary aim of the study was to develop a model of ECMO-based cadaver organ recovery in mice. Our model mimics uncontrolled organ donation after an “out-of-hospital” sudden unexpected death with subsequent “in-hospital” cadaver management post-mortem. The secondary aim was to assess blood gas parameters, cardiac activity as well as overall organ state. The study protocol included post-mortem heparin–streptokinase administration 10 min after confirmed death induced by cervical dislocation under full anesthesia. After cannulation, veno-arterial ECMO (V–A ECMO) was started 1 h after death and continued for 2 h under mild hypothermic conditions followed by organ harvest. Pressure- and flow-controlled oxygenated blood-based reperfusion of a cadaver body was accompanied by blood gas analysis (BGA), electrocardiography, and histological evaluation of ischemia–reperfusion injury. For the first time, we designed and implemented, a not yet reported, miniaturized murine hemodialysis circuit for the treatment of severe hyperkalemia and metabolic acidosis post-mortem.

**Results:**

BGA parameters confirmed profound ischemia typical for cadavers and incompatible with normal physiology, including extremely low blood pH, profound negative base excess, and enormously high levels of lactate. Two hours after ECMO implantation, blood pH values of a cadaver body restored from < 6.5 to 7.3 ± 0.05, pCO_2_ was lowered from > 130 to 41.7 ± 10.5 mmHg, sO_2_, base excess, and HCO_3_ were all elevated from below detection thresholds to 99.5 ± 0.6%, − 4 ± 6.2 and 22.0 ± 6.0 mmol/L, respectively (Student *T* test, *p* < 0.05). A substantial decrease in hyperlactatemia (from > 20 to 10.5 ± 1.7 mmol/L) and hyperkalemia (from > 9 to 6.9 ± 1.0 mmol/L) was observed when hemodialysis was implemented. On balance, the first signs of regained heart activity appeared on average 10 min after ECMO initiation without cardioplegia or any inotropic and vasopressor support. This was followed by restoration of myocardial contractility with a heart rate of up to 200 beats per minute (bpm) as detected by an electrocardiogram (ECG). Histological examinations revealed no evidence of heart injury 3 h post-mortem, whereas shock-specific morphological changes relevant to acute death and consequent cardiac/circulatory arrest were observed in the lungs, liver, and kidney of both control and ECMO-treated cadaver mice.

**Conclusions:**

Thus, our model represents a promising approach to facilitate studying perspectives of cadaveric multiorgan recovery for transplantation. Moreover, it opens new possibilities for cadaver organ treatment to extend and potentiate donation and, hence, contribute to solving the organ shortage dilemma.

**Supplementary Information:**

The online version contains supplementary material available at 10.1186/s40635-023-00534-2.

## Introduction

Donation after circulatory death (DCD) has emerged as a promising strategy for expanding the donor pool along with donation after brain death (DBD) (for review see [1]). With this regard, Maastricht category IA (individual found dead out-of-hospital) and IIA (out-of-hospital sudden unexpected irreversible cardiac arrest after unsuccessful resuscitation) of DCD patients represent the least studied category of donors with large transplantation potential [2, 3]. In this line, to provide an efficient organ supply for transplantation from this source of organs, adequate resuscitation methods are required. Since organs from DCD donors are particularly susceptible to the effects of warm ischemia injury, ECMO has been advocated as a preferred method for their maintenance and recovery. Hence, both hypothermic and normothermic abdominal regional perfusion by ECMO have gained increasing attention and prominence in recent years for in situ recovery and preservation of multiple organs in DCD donors [4–6]. However, reported transplantation outcomes from donors belonging to the Maastricht category I and II are limited typically to kidney and liver recovery using mentioned ECMO modes [7–11].

In heart transplantation, donors usually fall into Maastricht category III and are used as part of controlled DCD [12]. Of note, such cases involve ante-mortem cannulation for ECMO, withdrawal of life-sustaining therapy (WLST), circulatory arrest (5–10-min no-touch period) and ischemia time not exceeding 30 min [13, 14]. Therefore, to increase an organ yield per a DCD donor using ECMO, feasible experimental models of cadaver organ resuscitation (especially mimicking uncontrolled DCD) are necessary [3, 15–17]. Similar to clinical cases, a few large animal models reported on controlled DCD under normothermic extracorporeal support do not address post-mortem cardiac recovery (such as in Maastricht category I donors) and include ante-mortem cannulation and anticoagulation [18, 19]. In this regard, extracorporeal circulation models in small animals created prerequisites for studying uncontrolled DCD organ donation under ECMO [20]. On this account, our versatile mouse ECMO model opened new opportunities for its implementation in the field of in situ organ preservation and in particular permits exploring DCD organ recovery in a systematic way [21–23].

To our knowledge, neither clinical cases nor animal studies addressing heart recovery from DCD donors of Maastricht category I under ECMO after 1 h of warm ischemia in the absence of any ionotropic and cardioplegic treatments have yet been reported. Taken together, the overall aim of the study was to establish a murine model mimicking uncontrolled donation after unexpected circulatory death and a protocol for cadaver organ recovery using ECMO. The main steps of the protocol include: confirmed death after cervical dislocation, post-mortem anticoagulation, and fibrinolysis, 1 h of cadaver stay at room temperature (RT), post-mortem cannulation for V–A ECMO followed by ECMO perfusion for 2 h, and organ harvest. Furthermore, the assessment of ECMO-resuscitated cardiac activity using electrocardiography and the metabolic status of a cadaver body using BGA analysis was the secondary objective of the study.

## Materials and methods

### Animals and cadaver treatment

Mice of a C57BL6/J background were housed in the animal facility of the Centre for Operative Medicine of University Hospital Würzburg. Male and female animals aged 10 ± 1 months and with an average weight of 27 ± 5 g were sacrificed under full isoflurane anesthesia via cervical dislocation in accordance with the European Directive 2010/63/EU and German Animal Welfare Act (§4 TierSchG) [24]. The mice were assigned to two study groups (*n* = 5 each): ECMO and control animals. The sample size was chosen based on prior experience with murine ECMO. Irrespective of the anticipated high variability of data collected post mortem, in establishing the current protocol the selected sample size did not exceed the minimum number of experimental animals that was necessary to demonstrate statistical significance in metabolic parameters for live mice treated with ECMO [22]. All data generated in the present study conform to the ARRIVE guidelines (Animal Research: Reporting of In Vivo Experiments) [25].

After confirmation of death, murine cadavers were subjected to systemic anticoagulation by injecting into the right jugular vein 1000 international units (IU) of heparin (Ratiopharm GmbH, Germany) and preventive thrombolysis with 500 IU streptokinase (Sigma-Aldrich, Germany). Next, mice underwent moderate pressure closed-chest cardiac massage (CCCM) to mimic “unsuccessful resuscitation” and circulate the heparin and streptokinase for in total of 10 min at around 150 chest pressures per min. Mouse cadavers were connected to a custom-made miniaturized ECMO machine 1 h post-mortem using V–A-ECMO and circulated for 2 h followed by organ harvest (heart, liver, kidneys, and lungs). Control mice cadavers underwent the identical manipulations as experimental ones, except for undergoing extracorporeal circulation.

**Fig. 1:**
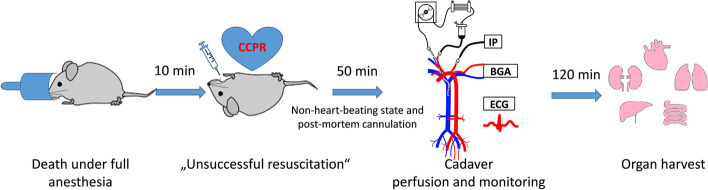
Schematic diagram of experimental design. *CCCM* stands for closed-chest cardiac massage, *IP* invasive pressure, *BGA* blood gas analysis, *ECG* electrocardiography

The general overview of the main experimental steps is presented in Fig. 1.

### Microsurgery, ECMO circuit, and physiological monitoring

Microsurgery, cannulation procedures, and connection to an ECMO machine were performed as previously described [21, 22] with the difference that instead of veno-venous ECMO, veno-arterial ECMO was performed in the present study. All microsurgical manipulations were performed under an operating Leica S6 D stereomicroscope (Leica Microsystems GmbH, Germany) in a plastic container placed on ice.

To expose the left *Arteria carotis (A. carotis)*, a short longitudinal skin incision was made along the anterior border of the sternocleidomastoid muscle. To avoid bleeding, bipolar coagulation of the small vessels was performed using Accu-Temp High Variable Temperature Cautery (BVI, USA). As a next step, *A. carotis* was separated from the vagus nerve and venous plexus. The distal part of the *A. carotis* was ligated with a 6-0 silk ligature. Then, the carotid lumen was carefully incised with microscissors, and an atraumatic FMC Fine Micro Cannula (Feeltech Co., Ltd, Korea) with a gauge size 25 G was advanced forwards until the aortic arch was reached. A cannula was separately blunted and polished for safe and minimally damaging handling. Afterward, the cannula was secured to a vessel by ligation. Blood that oozed out during surgery was retransfused into the ECMO circuit.

To expose the right *Vena jugularis,* a 4 mm lateral skin incision was performed on the right side of the neck. Once the vein has distally been ligated with 8–0 silk suture, a slip knot has been placed at the proximal end of the vein and its anterior wall was carefully incised. A flexible 0.6 mm polyurethane cannula was inserted into the proximal part of the jugular vein and gently pushed 2.5–3 cm toward the direction of the right atrium. Next, the cannula was affixed to the vein using 8–0 silk ligature. This cannula was constructed with multiple fenestrations to provide sufficient blood drainage from the right atrium and both the Vena cava superior and inferior.

**Fig. 2:**
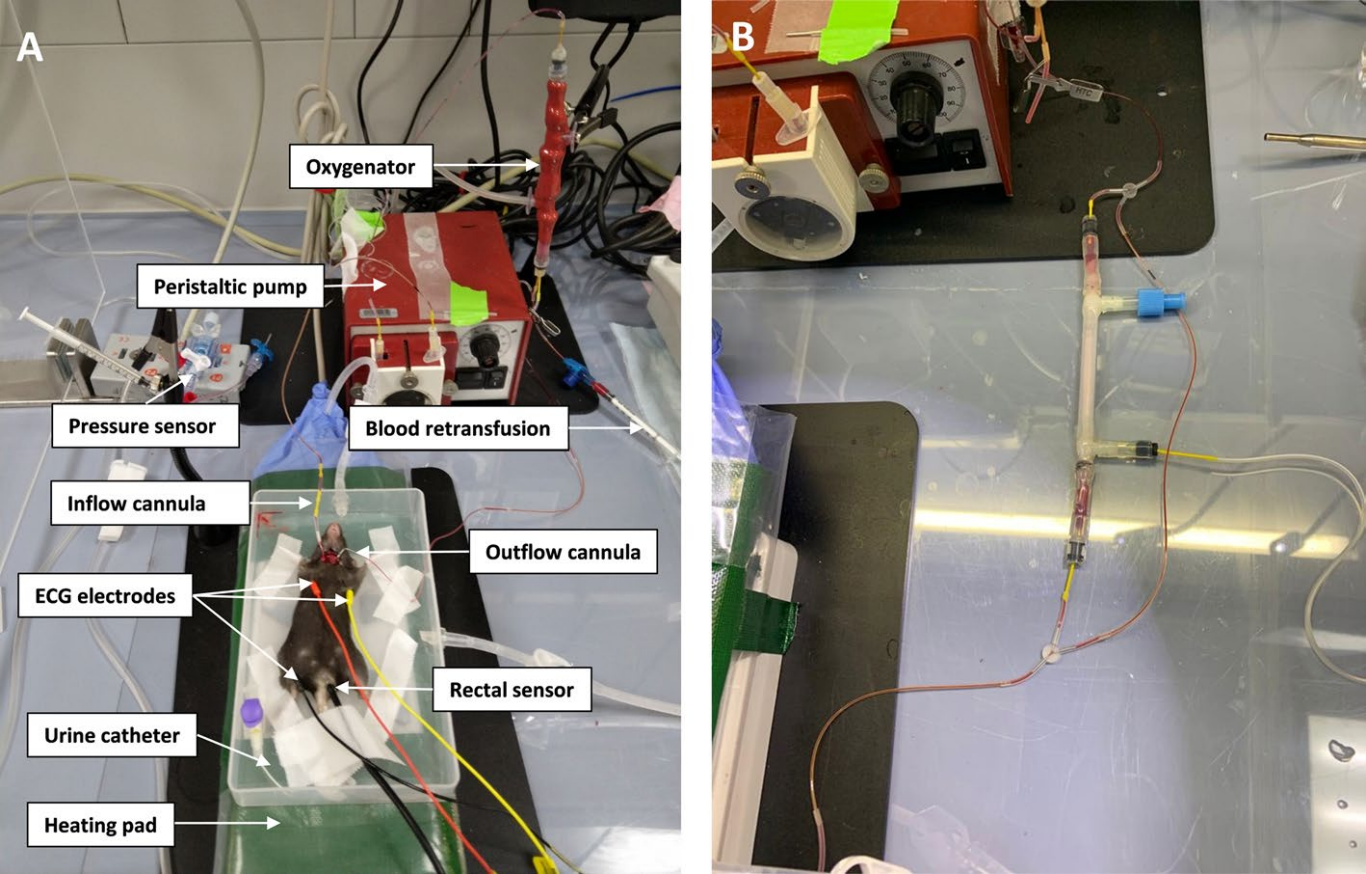
**A** Photo of the experimental setup used in cadaver mouse ECMO. The main components of the system are indicated with white arrows. Venous blood is drained by a peristaltic pump into the circuit via an inflow cannula, whereas newly oxygenated blood is returned to the systemic circulation via an outflow cannula. **B** Custom-made circuit for dialysis and ultrafiltration integrated into an ECMO murine circuit via Y-adaptors

The perfusion pressure was measured via a pressure transducer connected to an arterial cannula and maintained at the level of 50–70 mmHg. The pressure profile was simultaneously registered via a DPT-6000 pressure transducer (CODAN, Germany), an amplifier (Föhr Medical Instruments, Germany), and through a signal box (PowerLab 4/30, ADInstruments, United Kingdom) recorded on a computer (software: LabChart version 7, ADInstruments, United Kingdom). A custom-made miniaturized ECMO circuit was primed with 0.6 ml of 1:1 mixture of pre-oxygenated Krebs–Henseleit and 6% Volulyte solutions supplemented with 50 mM NaHCO_3_, 1.5 mM CaCl_2_, 1500 IU of heparin/250 IU streptokinase, 13.5 mM tris(hydroxymethyl)aminomethane (TRIS), 0.04 mg/ml of furosemide (pH of the priming solution 8.67). Every 45 min 150 µl of substitution buffer (the same as a priming solution but without heparin/streptokinase and Krebs–Henseleit solution, pH = 8.27) was injected into the circuit to compensate for intravasal liquid loss, and an acid/base disturbance. The general overview of the equipment used in our ECMO model for cadaver perfusion is depicted in Fig. 2 A for illustrative purposes. For the first time, we designed a hemodialysis circuit for mice with a priming volume of no more than 120 µl (Fig. 2B). For isovolemic removal of K^+^ ions and metabolic correction, a K^+^-free and bicarbonate-enriched multiBic® hemofiltration fluid was used. The total priming volume of the whole ECMO circuit was measured to be 620 µl, whereas the volume of the additional dialysis circuit amounted to only 120 µl. Two peristaltic 4-channel pumps (REGLO Digital, Ismatec, Germany) were utilized for propulsion within ECMO and dialysis circuits. To prevent clogging by tissue debris and thrombi, custom-made membrane oxygenators were equipped with a 70-µm cell filter. Mice cadavers were air-free connected to the primed circuit. The ECMO parameters used in this study were the following: blood flow rate 1.1–1.6 ml/min, O_2_ flow 500 mL/min, and FiO_2_ 100%. The flow rate used in a dialysis machine was 0.8 ml/ml. The partial pressure of oxygen (pO_2_) of a priming solution was monitored using a needle fiber-optic oxygen microsensor connected to a portable monitoring device (PreSens, Precision Sensing GmbH, Germany). The sensor was calibrated in accordance with the manufacturer's instructions.

On average, oxygenated perfusion in experimental cadaver mice was initiated 1 h post-mortem and they were circulated for 2 h.

### Blood gas analysis

Blood samples (70 µl) were collected from the right atrium (central venous blood) via a cannula inserted through a right jugular vein and a circuit directly after an oxygenator (arterial blood). To avoid excessive blood loss and hemodilution, BGA sampling was limited to a few timepoints: 60 min after death (starting reference data), 10 and 120 min after the ECMO initiation. The measurements were performed using a portable i-STAT Alinity point-of-care blood analyzer (Abbott Point of Care Inc., Princeton, USA). Before measurements, blood samples were pre-warmed in a thermostat set to 37 °C for 5 min. The following BGA parameters were assessed: the power of hydrogen (pH), partial pressure of carbon dioxide (pCO_2_), partial pressure of oxygen (pO_2_), bicarbonate (HCO_3_), base excess (BE), oxygen saturation (sO_2_) and total carbon dioxide (tCO_2_). In separate experiments, the concentration of K^+^ ions and lactate in arterial blood was determined.

### Electrocardiography

During ECMO of murine cadavers, electrocardiography records were continuously sampled via needle electrodes and recorded using a small animal physiological monitoring system (Harvard Apparatus, USA). ECG was collected in mice in a supine position atop a heating pad with subcutaneously affixed ECG electrodes as follows: right front paw, left front paw, and right hind limb. Electrocardiograms were denoised and smoothed using high and low frequency as wells Savitzky–Golay filters, respectively, in a Signal Processing Tool of MATLAB Software R2022b.

### Histopathological examinations

Organs were fixed in 4% paraformaldehyde (PFA, Morphisto, Germany) in phosphate-buffered saline (PBS, pH 7.4) for 2 h at room temperature at gentle shaking and transferred for additional incubation in freshly replaced PFA overnight at 4 °C. Paraffin-embedded organ fragments were routinely processed and cut longitudinally (liver, kidney, lungs) and in transverse sections (heart) at 5 μm thickness with a sliding microtome (SM2010 R, Leica, Germany). Sections were stained with hematoxylin and eosin (H&E) and assessed for histopathological changes by a qualified pathologist. Images were acquired using a PANNORAMIC SCAN II Digital Slide Scanner (3DHISTECH Ltd., Hungary) and evaluated using imaging software CaseViewer version 2.4.

### Statistics

Data were analyzed for normal distribution using the Shapiro–Wilk test. Student *T* test with a significance level of *p* < 0.05 was used for the statistical analysis. Data are presented as mean ± standard deviation (SD). Statistical analyses were performed using IBM SPSS Statistics version 26.0 software (IBM Corp. Armonk, NY, USA).

## Results

### Establishment of a protocol for cadaver organ recovery using ECMO in a murine model of uncontrolled donation after unexpected circulatory death

A protocol for cadaver organ resuscitation in a murine model of uncontrolled DCD donation mimicking Maastricht subcategories IA and IIA in humans (according to the modified Maastricht classification of DCD in Paris 2013 [2]) was established. In this scenario, cannulation and organ treatment with ECMO can generally begin once a cadaver body arrives at the hospital. For that, mice with a C57BL6/J background (*n* = 5, age 10 ± 1 months, weight 27 ± 5 g) were included in the study. The age of mice was deliberately selected to reproduce the situation in the human adult population with an age of 40–50 years when the incidence of sudden death is most frequent and organ quality for transplantation is relatively high. To reproduce unexpected circulatory death, mice were sacrificed using cervical dislocation. After confirmed death, murine cadavers were subjected to systemic anticoagulation with heparin and preventive thrombolysis with streptokinase. After “unsuccessful resuscitation” mice were left for 1 h at RT (22–24 °C). To recover cadaver organs, murine cadavers were connected via V–A cannulation topography to a murine ECMO machine. No cardioplegia and cardioprotection was used in our protocol. ECMO circuit was specifically primed with an alkaline solution containing TRIS and NaHCO_3_ as well as hydroxyethyl starch to combat post-mortem acidosis, edema, and intravasal liquid loss. In our experimental settings, the body temperature of murine cadavers in around 1 h under ECMO raised gradually to and was kept at 34 °C. Organs recovered for 2 h under ECMO were subsequently harvested. Another feature of our model is the design and implementation of murine hemodialysis as a part of a protocol to treat hyperkalemia and hyperlactatemia. In contrast to living animals, apparently, a rapid and complete loss of vascular myogenic tone is expected in mouse cadavers shortly after cervical dislocation. In our model, we used low flow rates (1.1–1.6 ml/min) to avoid overperfusion and provide some protection against shear stress to the vessels, still having postmortem blood clots while not impending metabolic exchange. One another highlight in our model is the combined post-mortem use of heparin and streptokinase to prevent further thrombosis and start intravascular thrombolysis.

### Assessment of blood gas parameters

To obtain baseline BGA values, venous blood samples taken directly from the right atrium were subjected to BGA analysis following 1-h post-mortem prior to extracorporeal circulation. According to the BGA data, pronounced acidosis, hypoxemia, and hypercapnia in murine cadavers were manifested by initially low pH (< 6.5) accompanied by low pO_2_ (44.7 ± 24.8 mmHg) and high pCO_2_ (> 130 mmHg), respectively (Table 1). Within 10 min after ECMO initiation, some improvements in all tested BGA parameters were observed. Specifically, in venous blood pH shifted to 7.0, pO_2_ increased to 50.3 ± 5.1, whereas pCO_2_ decreased to 105.4 ± 20.1 mmHg, accordingly. At this timepoint, initially undetectable central venous saturation (ScvO_2_) increased to 58.0 ± 4.2%, base excess was − 4.5 mmol/L, HCO_3_ corresponded to 26.8 ± 6.9 mmol/L and tCO_2_ reached 30.0 ± 7.1 reflecting a tendency toward partial stabilizing of ischemia-compromised acid–base balance. Similar data were obtained for arterial blood. Combined treatment with trometamol-containing substitution solution and continuous oxygenation allowed for BGA parameters to almost reach physiological values by 120 min of ECMO treatment. Specifically, in arterial blood pH was measured to be 7.3, sO_2_ reached 99.5. ± 0.6%, while pCO_2_ further declined down to 41.7 ± 10.5 and pO_2_ increased up to 156.6 ± 63.5 mmHg, respectively. Moreover, HCO_3_ was detected to be 22.0 ± 6.0, BE was − 4 ± 6.2 and tCO_2_ corresponded to 23.2 ± 6.6 mmol/L, respectively. In addition, ScvO_2_ elevated further to 91% at the end of the ECMO treatment. In addition, we measured also lactate and potassium levels in the course of ECMO as important ischemia-associated indicators. In contrast to the above parameters, their concentration in blood remained unalterably high: > 20 for lactate and > 9 mmol/L for K^+^, respectively. Therefore, to overcome this dilemma, we designed and implemented a fully functioning novel mouse model of hemodialysis integrated into a cadaver ECMO circuit. To our knowledge, no hemodialysis circuit for mice has yet been reported. Hemodialytic treatment based on K^+^-free solution was started 30 min following ECMO initiation. After 60 min under hemodialysis K^+^ levels decreased to 6.9 ± 1.0, whereas lactate levels decreased to 10.5 ± 1.7 mmol/L, correspondingly.

**Table 1:** BGA findings

Blood type and collection site	Collection time	pH	pCO_2_, mmHg	pO_2_, mmHg	HCO_3_, mmol/L	BE, mmol/L	sO_2_, %	tCO_2_, mmol/L	K^+^, mmol/L	Lactate, mmol/L
Central venous (right atrium)	60 min post-mortem	< 6.5	> 130	44.7 ± 24.8	ND	ND	ND	ND	> 9	> 20
Central venous (right atrium)	10 min after ECMO initiation	7 ± 0.1	105.4 ± 20.1	50.3 ± 5.1	26.8 ± 6.9	− 4.5	58.0 ± 4.2	30.0 ± 7.1	> 9	> 20
Arterial (post-oxygenator)	10 min after ECMO initiation	6.9 ± 0.2	87.2 ± 30.5	83.5 ± 20.1	20.6 ± 12.6	− 12.0 ± 15.7	83.3 ± 4.6	23.3 ± 13.5	> 9	> 20
Arterial (post-oxygenator)	120 min after ECMO initiation	7.3 ± 0.05*	41.7 ± 10.5*	156.6 ± 63.5*	22.0 ± 6.0	− 4 ± 6.2	99.5 ± 0.6*	23.2 ± 6.6	> 9	> 20
Arterial (post-oxygenator)	120 min after ECMO initiation (90 min on dialysis)								6.9 ± 1.0	10.5 ± 1.7

*ND* not detectable

*Differences are significant at *p* < 0.05 compared to arterial blood samples (10 min)

### Electrocardiography outcomes

**Fig. 3:**
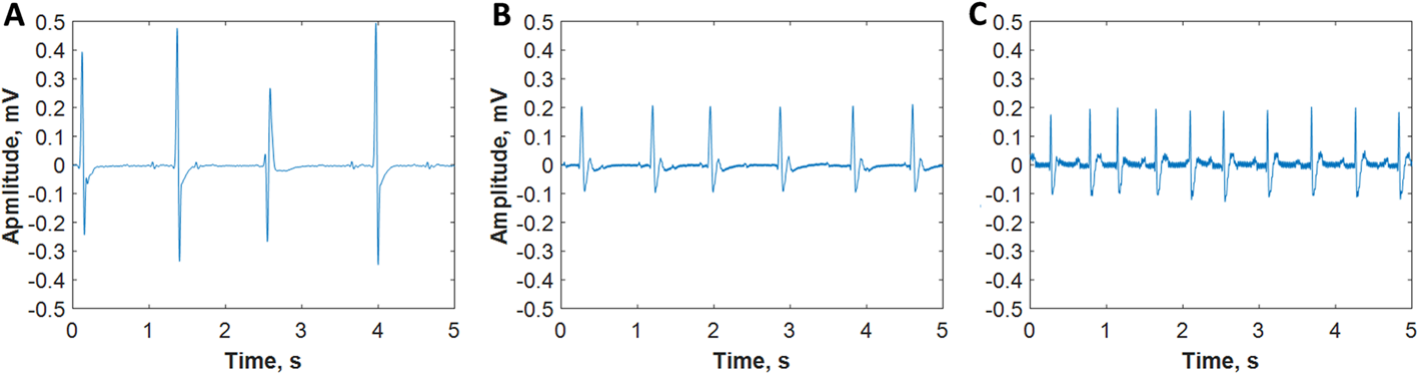
Exemplary sequence of ECG recordings acquired in mice post-mortem: heart re-beating 8 min after ECMO initiation (**A**) and heart recovery before (**B**) and 60 min after dialytic treatment (**C**)

As confirmed after death, there was no electrical activity in ECGs prior to the starting of the ECMO. The first signs of the post-mortem heart re-beating under ECMO were detected on average after 8–10 min. In all experiments, the starting ECGs were characterized by severe bradycardia, with the heart rates being varied in the range of 78 ± 21 bpm. As ECMO support progressed, the heart rate increased up to 156 ± 27 bpm (1 h ECMO). In the absence of hemodialysis, the analysis of ECGs revealed the following common changes in ECG peaks: absence of p-waves, T-wave inversions, ST-segment depression, and elevation as well as broadening of QRS complexes. According to ECG data, after the initiation of hemodialysis marked signs of myocardial recovery (sinus rhythm, normal QRS morphology, no alterations in the ST segment, Fig. 3C) followed initially pronounced bradycardia and arrhythmia (Fig. 3A, B).

Opening of the thoracic cavity after a 2-h course of the ECMO demonstrated active heart beating (see Additional file 1: Video S1).

### Histological evaluation of organ damage

**Fig. 4:**
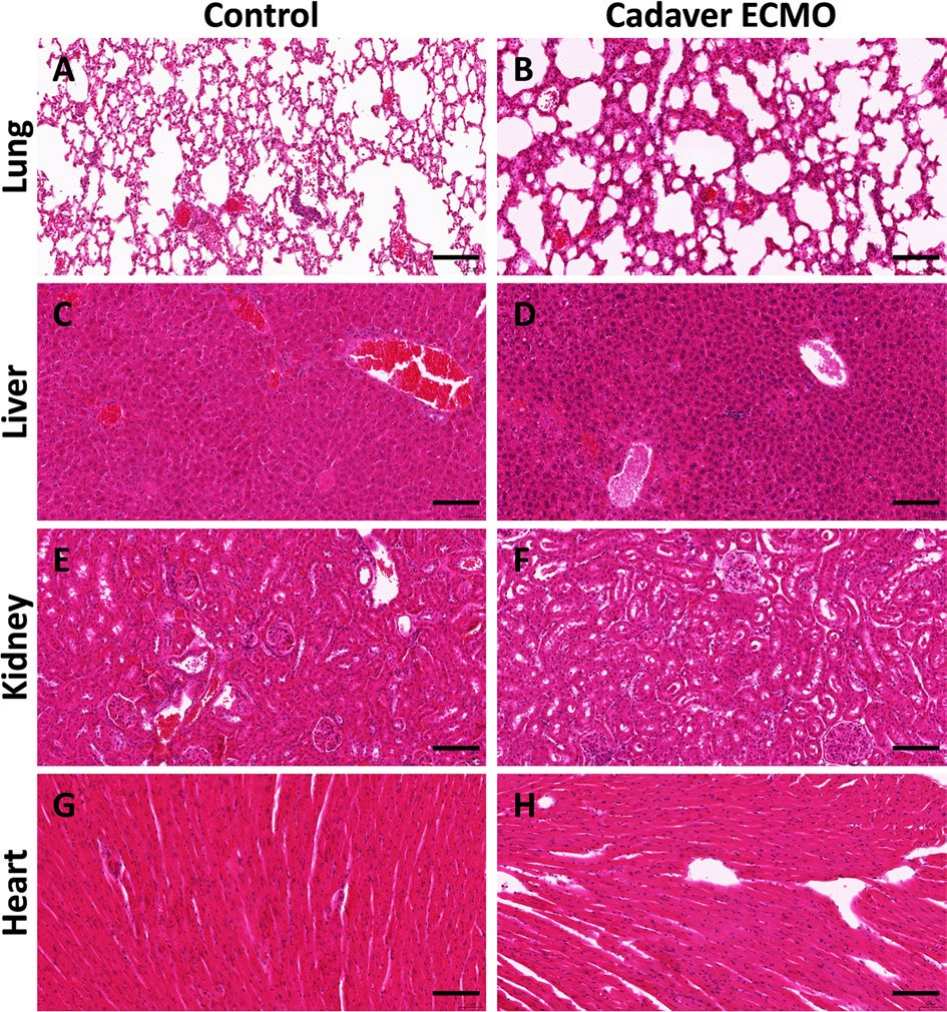
Representative histological sections of the indicated organs: lung (**A**, **B**), liver (**C**, **D**), kidney (**E**, **F**), and heart (**G**, **H**) stained with H&E. Left panel depicts specimens taken from control animals (3-h post-mortem, no perfusion) and right panel demonstrates ECMO animals. The scale bar is 100 µm

Figure 4 shows representative histology of organs after a 2-h ECMO treatment and control organs from animals that have been dead for the same timespan without reperfusion. We focused on organs that are typically transplanted in humans: lungs, liver, kidneys, and heart. The lungs of mice subjected to ECMO revealed dystelectasis, marked interstitial edema, hemorrhage, and congestion (Fig. 4B) in comparison with untreated cadavers (Fig. 4A). In contrast, we did not observe readily apparent microscopic differences in the liver (Fig. 4D vs. C) and the kidneys (Fig. 4F vs. E). However, both organs displayed signs of congestion that were not overtly different in both cohorts. Importantly, liver cell necrosis or tubular necrosis of the kidneys was not evident. The myocardium of both groups was unremarkable (Fig. 4H, G).

## Discussion

Our current experimental setup with cervical dislocation and 1 h warm ischemia serves as a model of unwitnessed sudden death outside of the hospital and mimics uncontrolled DCD donation such as according to the Maastricht subcategories IA and IIA in humans (according to the modified Maastricht classification of DCD in Paris 2013) [2]. Our protocol for cadaver organ resuscitation encompasses anticoagulation and fibrinolysis of a cadaver body followed by V–A-ECMO enhanced with hemodialysis. To our knowledge, no papers addressing ECMO- and hemodialysis-supported DCD organ resuscitation in small animals with the mentioned categories of potential donors have been published, making this a novel model.

Results of blood gas analysis indicate that during the 120-min ECMO-assisted reperfusion oxygenation parameters significantly improved and almost returned to physiological levels. At the same time, lactate, and circulating K^+^ levels remained high. Notably, metabolic breakdown of lactate requires prolonged time, and K^+^ accumulation (most probably due to hemolysis) is associated with increased risk of adverse consequences Given that cadaver organs are impaired by extreme ischemic conditions, hemodialysis support was necessary. Therefore, we used hemodialysis to ameliorate lactic acidosis and hyperkalemia. Since samples used in postmortem biochemical analysis are always affected by hemolysis, the concentration of lactate and potassium was measured after ultrafiltration of hemolyzed arterial blood [26]. Following hemodialysis, partial lactate and potassium clearance was observed which is in correspondence with the literature data [27].

In this study, we placed our focus primarily on post-mortem heart recovery, since the restoration of its contractile properties is crucial for the recovery of other organs, and the DCD heart is very susceptible to hypoxemia and ischemic damage Observed adverse alterations in DCD heart activity could be attributed to blood hyperkalemia (for review see [28]). Implementation of hemodialysis and ultrafiltration allowed to reduce the concentration of K^+^ ions and consequently improve cardiac contractility. Notwithstanding, histological examination of cardiac tissues from both control and experimental mice identified no apparent pathological alterations in the heart. It might be partially explained by the short observation period for histological changes to occur in hearts subjected to 2-h ECMO after 1-h post-mortem ischemia. The pathogenesis of the observed changes in the lungs is likely multifactorial. The observed dystelectasis in both control and ECMO cohorts of mice very likely reflected the complications of the absent mechanical ventilation. For effective gas exchange to occur, alveoli must be sufficiently ventilated and perfused. Continued perfusion of congested lungs is expected to rapidly elevate the hydrostatic pressure in the pulmonary microvasculature. As a result, similar to what we have observed in our model, compensatory pulmonary edema occurs and might diminish the gas exchange [29]. Furthermore, such complications are expected to be exacerbated by post-mortem loss of vasculature tone, congestion as well as microthrombi formation (despite initial fibrinolytic treatment) in the pulmonary vascular system. This leads to disturbance of the endothelial barrier and increased flow resistance. All mice in this study were sacrificed by cervical dislocation which is known to cause artificial disturbances of lung histology. These include congestion of alveolar capillaries and interstitial edema [30]. However, since the histopathological alterations were more marked in the ECMO-treated cohort, we believe that the observed changes are not a simple consequence of cervical dislocation, but represent a true effect of post-mortem ECMO.

**Fig. 5:**
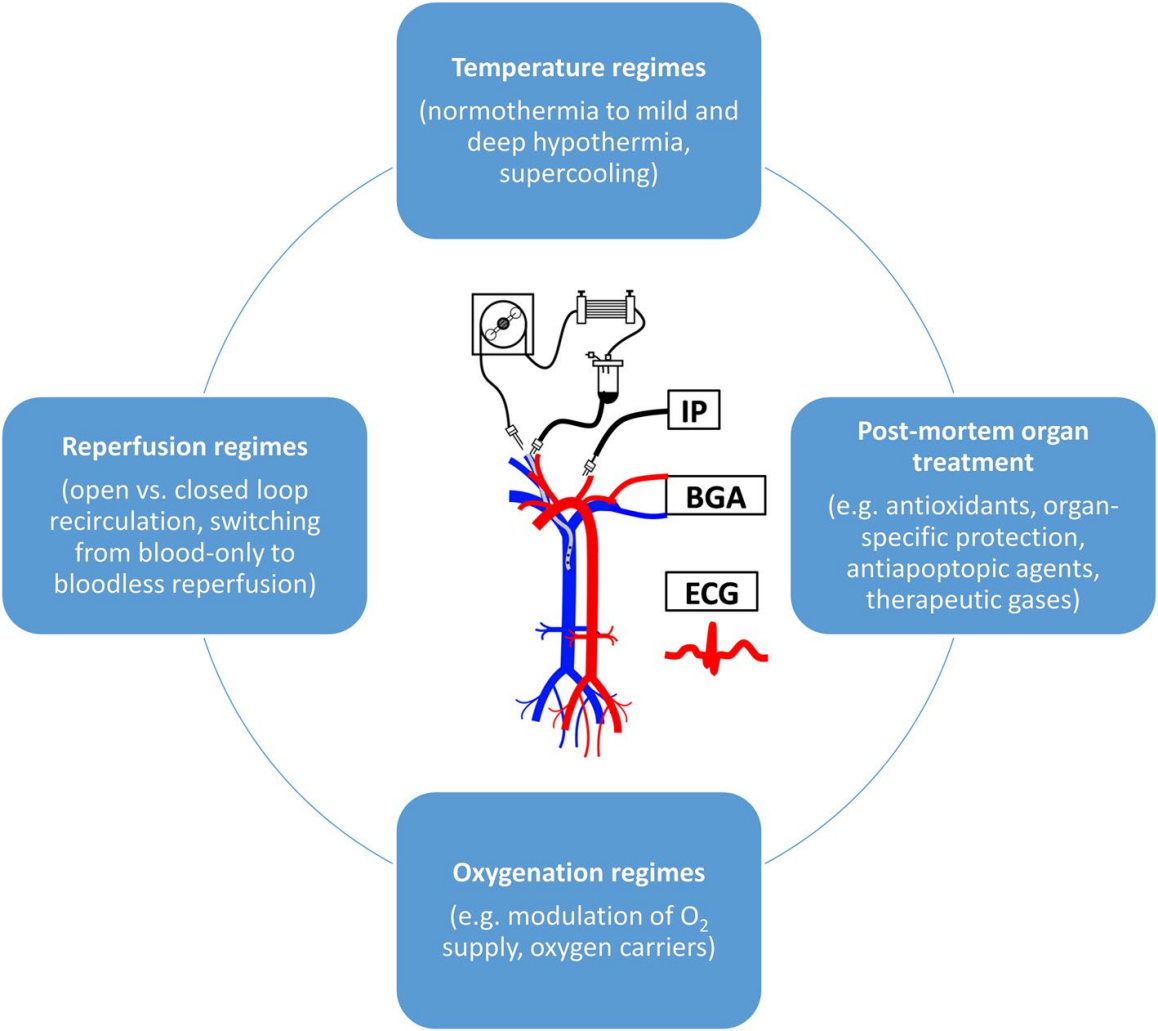
Suggested future strategies toward increasing in situ cadaver organ shelf-life under ECMO and oxygenated perfusion

The availability of ECMO as an intensive care treatment in hospitals is an essential prerequisite for effective post-mortem support of multiple potential organs for donation [31–34]. A cadaver state is linked to marked physiological instability; therefore, adequate cadaver management has to be attempted to prevent deterioration in organ function before transplantation [35, 36]. Our murine model allows testing a wide spectrum of post-mortem organ treatments, e.g., for optimization of temperature regimes, perfusion solution formulations, and application of therapeutic gases. Among potential targets, various anti-ROS, anti-apoptotic, and organ-specific protective agents can be systematically tested for cadaver systemic treatment [37]. Separate research efforts in future studies might be dedicated to optimizing oxygenation using oxygen carriers [38]. In the context of the present work, there are various preclinical murine models related to transplantation available. Murine transplant models with cardiomyopathies [39], end-stage kidney [40], and pulmonary diseases [41] or cystic fibrosis [42] are experimentally established. This potentially enables our model recapitulation of a whole plethora of clinical conditions and transplantation scenarios. From this standpoint, large-animal and even rat models seem to be so far lesser abundant and robust. Moreover, we believe that low-flow perfusion as used in the study could be an option for extending the organ shelf life by preventing energy depletion and reducing edema as well as damage to vascular endothelium. For illustrative purposes, Fig. 5 features improvement strategies that could be directed toward optimizing cadaver ECMO protocols.

The present study accounts for the following limitations. First, our model necessitates well-established microsurgical skills. Second, although the model is potentially clinically translatable but still the transplantation models including knock-in/out recipients and experimental protocols widely used for mimicking solid organ transplantation (e.g., Langendorff model for the heart) or even heterotopic murine heart transplantation have to be implemented in forthcoming studies. Furthermore, complementary research in larger nonrodent mammalian species might offer several advantages, such as enhanced surgical and endoscopic accessibility and scientifically justifiable xenotransplantation studies. Third, we examined the effects of cadaveric ECMO on organ recovery out to a pre-determined 120 min endpoint. Extending the duration of ECMO reperfusion would yield more comprehensive insights into the opportunities for ECMO-assisted recovery of DCD organs. Fourth, in our experimental conditions mice were not intubated to specifically assess the contribution of pure ECMO perfusion rather than mechanical lung ventilation to overall tissue oxygenation. Therefore, this aspect has to be taken into consideration for future experiments focusing on lung recovery and preservation under ECMO [4]. In the post-mortem blood-mixed reperfusion of the DCD cadaver body, the rate of blood hemolysis as well as accumulation of pro-inflammatory cytokines are anticipated to progressively increase over time. In the present context, bloodless reperfusion modes might provide a good alternative method for organ recovery.

## Conclusions

The pretransplant in situ post-mortem multiorgan recovery with ECMO is a promising approach for extending the organ pool, prolonging organ shelf life, and thus helping to combat the organ scarcity crisis. As a result of cadaveric ECMO perfusion and metabolic correction, heart contractile properties and metabolic parameters were significantly improved. Moreover, we have implemented our novel, a not yet reported, murine hemodialysis integrated into the ECMO circuit to correct extremely altered metabolic state after death. Thus, our approach enables the implementation of a variety of modern blood purification methods in cadaveric organ recovery using extracorporeal life support. For this reason, our model opens up novel therapeutic options for cadaver organs, thereby increasing the available organ supply and prolonging preservation time prior to transplantation.

## Supplementary Information

**Additional file 1. **The video demonstrates recovery of heart beating in 2 h after ECMO initiated 1 h post-mortem.

## Data Availability

The data sets generated during and/or analyzed during the current study are available from the corresponding author upon reasonable request.

## Abbreviations

ECMO: Extracorporeal membrane oxygenation
V–A ECMO: Veno-arterial extracorporeal membrane oxygenation
BGA: Blood gas analysis
ECG: Electrocardiogram;
Bpm: Beats per minute
SCD: Sudden cardiac death
CVDs: Cardiovascular diseases
CCCM: Closed-chest cardiac massage
PBS: Phosphate-buffered saline
PFA: Paraformaldehyde
TRIS: Tris(hydroxymethyl)aminomethane
IU: International units
UW solution: University of Wisconsin solution
HTK solution: Histidine–tryptophan–ketoglutarate solution
H&E staining: Hematoxylin and eosin staining
SD: Standard deviation
WLST: Withdrawal of life-sustaining therapy
